# PECARN Rule in diagnostic process of pediatric patients with minor head trauma in emergency department

**DOI:** 10.1007/s00431-022-04424-9

**Published:** 2022-02-22

**Authors:** Alessandro Gambacorta, Marianna Moro, Antonietta Curatola, Federica Brancato, Marcello Covino, Antonio Chiaretti, Antonio Gatto

**Affiliations:** 1 Dipartimento Di Pediatria, Fondazione Policlinico Universitario “Agostino Gemelli”, IRCCS, Università Cattolica del Sacro Cuore, Rome, Italy; 2 Dipartimento Di Pediatria, Università Cattolica del Sacro Cuore, Rome, Italy; 3 Dipartimento Di Medicina d’Emergenza, Fondazione Policlinico Universitario A. Gemelli IRCCS, Università Cattolica del Sacro Cuore, Rome, Italy; 4 Dipartimento Di Pediatria, Fondazione Policlinico Universitario “Agostino Gemelli”, IRCCS, Rome, Italy

**Keywords:** PECARN Rule, Head injury, Pediatric, Emergency department, Personalized medicine

## Abstract

This study aims to evaluate the efficacy of the PECARN Rule (PR) in reducing radiological investigations in children with mild traumatic head injury in comparison with current clinical practice. A retrospective study was performed in our hospital between July 2015 and June 2020. Data of all children < 18 years of age admitted to the emergency department (ED), within 24 h after a head trauma with GCS ≥ 14, were analyzed. PECARN Rule was retrospectively applied to all patients. In total, 3832 patients were enrolled, 2613 patients ≥ 2 years and 1219 < 2 years. In the group of children ≥ 2 years, 10 presented clinically important traumatic brain injury (ciTBI) and were hospitalized, 7/10 underwent neurosurgery, and 3/10 clinical observation in the pediatric ward for more than 48 h. In children < 2 years, only 3 patients presented ciTBI, 2 underwent neurosurgery and 1 hospitalized. Applying the PR, no patient with ciTBI would have been discharged without an accurate diagnosis and we would have avoided 139 CT scans in patients ≥ 2 years, and 23 in those < 2 years of age (29% less).

*Conclusion*: We demonstrated the safety and validity of the PR in our setting with 100% sensitivity in both age groups in identifying patients with ciTBI and theoretically in reducing performed CT scans by 29%. Therefore, in patients classified in the low-risk category, it is a duty not to expose the child to ionizing radiation.

**Table:** 

**What is Known:** *• CT is the gold standard to identify intracranial pathology in children with head injury but CT imaging of head-injured children expose them to higher carcinogenic risk.* *• PECARN Rules support doctors in identifying children with ciTBI in order to reduce exposure to ionizing radiation.*	
**What is New:** *• We demonstrate the safety and validity of the PR with 100% sensitivity in both age groups in identifying patients with ciTBI.* *• In our setting, the application of PECARN Rule would theoretically have allowed us to reduce the CT scan by 29%.*

**Supplementary Information:**

The online version contains supplementary material available at 10.1007/s00431-022-04424-9.

## Introduction

Head injury is the leading cause of death and disability in children and a frequent reason of evaluation in the pediatric emergency department (ED) [1]. More than 90% are minor head injuries (MHI), rarely associated with brain injury or long-term sequelae [2, 3]. Only in a few cases pediatric patients with MHI present a clinically important traumatic brain injury (ciTBI) [4]. CT scan is the gold standard for the evaluation and management of patients with head trauma; it is highly sensitive in identifying clinically significant brain lesions that require acute intervention [5]. The clinical challenges in evaluating MHI in pediatric patients are as follows: identifying children with ciTBI in order to limit unnecessary exposure to carcinogenic ionizing radiation, decreasing the need for sedation for non-compliant patients, and reducing costs.

The Pediatric Emergency Care Applied Research Network (PECARN) Head Injury Decision Rule (PR) is an age-based rule published in 2009 by the Pediatric Emergency Care Applied Research Network. This rule can be implemented for children younger and older than 2 years of age to identify those at low risk for ciTBI, so that CT scan can be safely avoided. Kupperman et al. developed this clinical rule from a very high number of patients younger than 18 years, presenting within 24 h of head trauma, with Glasgow Coma Scale (GCS) scores of 14–15, in 25 North American emergency departments [6] (Suppl. Figure 1). It was subsequently validated in other populations and settings [7, 8]. PR is currently used in many countries for the ED management of children with MHI [9–11].

The aims of our study were to evaluate the efficacy and safety of the PR in detecting the presence of ciTBI, to reduce radiological investigations, and to quantify the margins for improvement in comparison with the current clinical practice in an ED setting. The secondary objective was to predict, with the PR, the incidence of ciTBI and/or fractures on CT scan even if not clinically important, in patients who underwent a head CT scan.

## Methods

### Study design and patients

We performed a retrospective observational study of children with MHI admitted to the ED of A. Gemelli Hospital in Rome, with annual attendance of about 13,000 patients younger than 18 years of age. The time period between July 2015 and June 2020 was retrospectively analyzed. We included children less than 18 years of age presenting to the ED within 24 h of head trauma with GCS ≥ 14.

During the study period, there was no specific internal protocol, the different clinicians independently used various guidelines on this topic reported in the literature. We excluded children with severe head trauma; those with trauma that occurred more than 24 h before the emergency room visit; patients who performed neuroimaging in another hospital or had only come for counselling from another hospital; patients who did not wait for the evaluation or refused clinical observation; and patients who lacked the necessary data for the application of the PR.

### Clinical protocol

In our analysis, we applied the PR to all children enrolled distinguishing two subpopulations of children over and under 2 years old as required by the rule. Thereafter, we distinguished patients according to the three categories of recommendations of the PR: recommended CT, CT versus observation, and CT not recommended (Suppl. Figure 1).

We defined, in accordance with Kuppermann [6], clinically important traumatic brain injury (ciTBI) by any of the following descriptions: death from traumatic brain injury; neurosurgical intervention for traumatic brain injury; intubation of more than 24 h for traumatic brain injury; hospital admission of 2 nights or more for the traumatic brain injury in association with traumatic brain injury on CT.

We defined “abnormal CT findings” any lesion visible at the CT scan involving the brain or the skull that does not fall within the definition of ciTBI.

### Data collection

Patients were identified from the hospital computerized clinical record (GIPSE®) by searching for the keywords “head injury”, “concussion”, “head trauma”, and “road injury” for all patients admitted to the ED.

Clinical and demographic data were collected by means of a form specifically developed for the study by pediatric specialists after being trained in data collection (Table 1).

**Table 1:** Clinical and demographic characteristics of the study population

Characteristics	Study cohort (*N* = 3832)	< 2 years (*N* = 1219)	≥ 2 years (*N* = 2613)
**Median age (years)**	5.3 (SD 4.8)	0.99 years (IQR 0.63–1.39)	6.01 (IQR 3.44–10.73)
**Gender (male)**	2381 (65.13%)	665 (54.6%)	1716 (65.6%)
**Time in PED (hours)**	2.42 (IQR 1.15–3.78)	2.43 (IQR 1.27–3.62)	2.4 (IQR 1.30–3.83)
**Re-visit in ED**	46 (1.2%)	12 (0.98%)	34 (1.3%)
**Triage color code** ** Red** ** Yellow** ** Green**	47 (1.2%) 726 (19%) 3059 (79.8%)	2 (0.17%) 203 (16.65%) 1014 (83.18%)	45 (1.72%) 523 (20.02%) 2045 (78.26%)
**Trauma site** ** Frontal** ** Parietal** ** Temporal** ** Occipital** ** Facial** ** Unknown**	1384 (36.1%) 419 (10.9%) 217 (5.7%) 774 (20.2%) 197 (5.1%) 1130 (29.5%)	477 (39.1%) 126 (10.3%) 56 (4.6%) 151 (12.4%) 36 (3%) 441 (36.2%)	907 (34.7%) 293 (11.2%) 161 (6.16%) 623 (23.8%) 161 (6.2%) 689 (26.4%)
**Transportation** ** Ambulance** ** Helicopter rescue** ** Own vehicles**	586 (15.3%) 30 (0.8%) 3216 (83.9%)	110 (9%) 8 (0.7%) 1101 (90.3%)	476 (18.2%) 22 (0.8%) 2115 (81.0%)
**Mechanism of injury** ** Domestic injury** ** Motor vehicle accident** ** School accident** ** Sports-related** ** Aggression** ** Other accidents** ** Undefined**	1834 (47.9%) 331 (8.6%) 368 (9.6%) 198 (5.1%) 23 (0.6%) 643 (16.8%) 435 (11.4%)	895 (73.4%) 46 (3.8%) 27 (2.2%) 0 0 135 (11.1%) 116 (9.5%)	939 (35.9%) 285 (10.9%) 341 (13.1%) 198 (7.6%) 23 (0.9%) 508 (19.4%) 319 (12.2%)
**Destination** ** Discharged at home** ** Hospitalization in the ward**	3680 (96%) 152 (4%)	1168 (95.8%) 51 (4.2%)	2512 (96.1%) 101 (3.9%)
**CT scan** ** Not performed** ** Performed** ** Abnormal**	3281 (85.6%) 551 (14.4%) 89 (2.3%)	1123 (92.1%) 96 (7.9%) 49 (4.0%)	2158 (83.6%) 455 (17.4%) 40 (1.5%)
**CT scan not performed according with PR recommendations** ** CT recommended** ** CT versus observation** ** CT not recommended**	2/3281 (0.06%) 669/3281 (20.4%) 2610/3281 (79.54%)	1/1123 (0.1%) 302/1123 (26.9%) 820/1123 (73.0%)	1/2158 (0.05%) 367/2158 (17%) 1790/2158 (82.95%)
**CT scan performed according with PR recommendations** ** CT recommended** ** CT versus observation** ** CT not recommended**	45/551 (8.1%) 342/551 (62.1%) 164/551 (29.8%)	16/96 (16.7%) 56/96 (58.3%) 24/96 (25.0%)	29/455 (6.4%) 286/455 (62.8%) 140/455 (30.8%)
**Abnormal CT scan according with PR recommendations** ** CT recommended** ** CT versus observation** ** CT not recommended**	13/89 (14.6%) 74/89 (83.2%) 2/89 (2.2%)	8/49 (16.3%) 40/49 (81.7%) 1/49 (2.0%)	5/40 (12.5%) 34/40 (85.0%) 1/40 (2.5%)
**ciTBI**	13 (0.3%)	3 (0.2%)	10 (0.4%)
**Neurosurgical intervention**	9 (0.2%)	2 (0.16%)	7 (0.3%)

### Data analysis

For children undergoing brain CT, we also analyzed any visible lesion involving the brain or the skull; neurosurgery need; hospitalization time in days; and site of the head injury.

### Statistical analysis

Categorical variables are reported as percentages. Continuous variables are described using medians and interquartile ranges (IQR). We performed comparisons between groups by means of chi-squared tests for categorical variables. Parameters displaying *p* < 0.05 were considered statistically significant. Sensitivity, specificity, and ROC area under the curve (AUC) are presented as values (95% confidence interval). We performed multivariate logistic regression models including variables with a *p* value < 0.05 in the univariate analysis.

## Results

During the study period, the medical records of 4943 patients were retrospectively analyzed. We considered 3832 patients eligible for the study (mean age 5.3 years, SD 4.8), 2613 patients were ≥ 2 years, while 1219 were < 2 years.

All the demographic data of the study population, the time spent in the pediatric ED, the need for a re-visit, the mechanism of trauma, the site of the trauma, and the triage code, are summarized in Table 1 and divided into age groups.

**Fig. 1:**
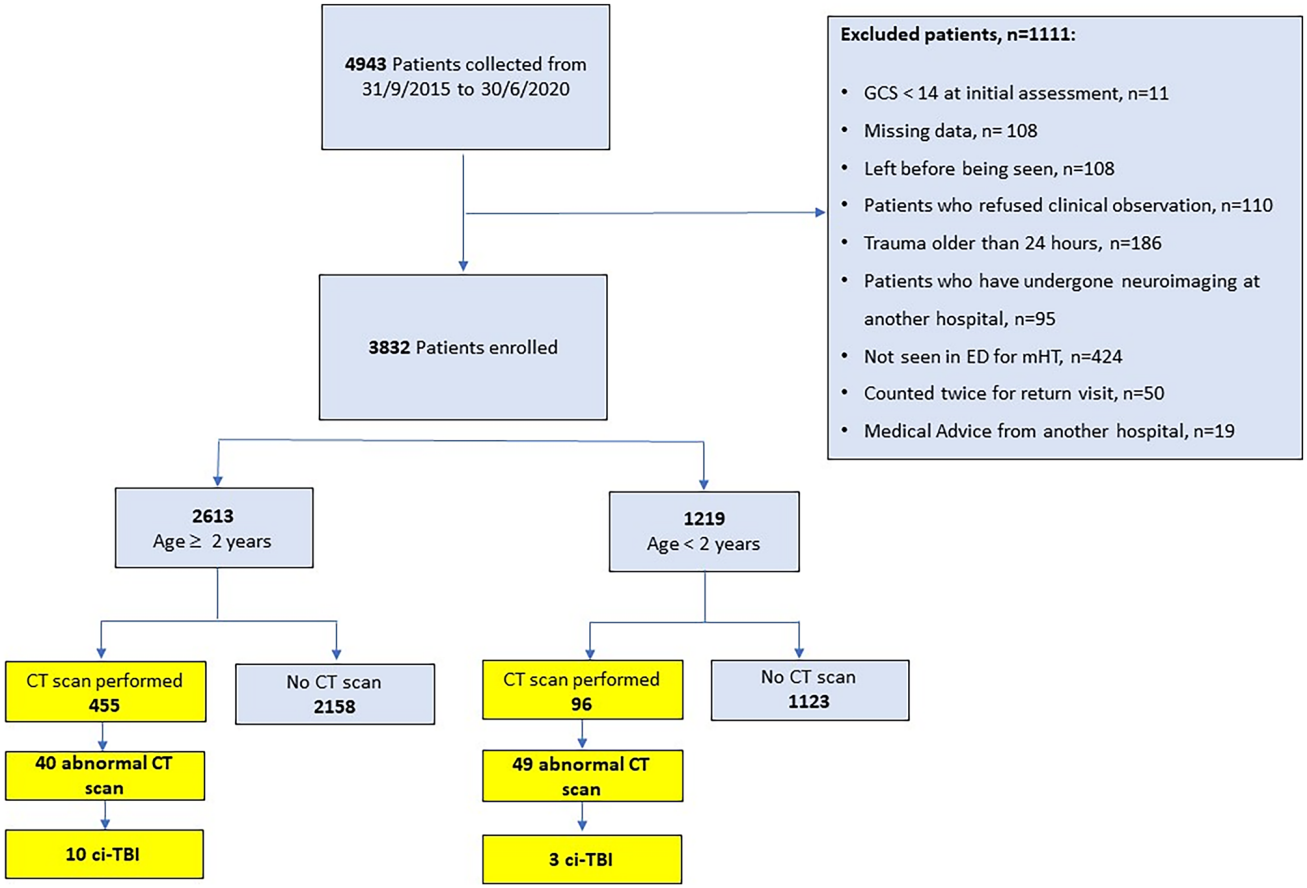
Flow chart of study

Two years and older-We included 2613 patients aged 2 years or older, of whom 455 (455/2613, 17.4%) received a CT scan, 40/455 (8.8%) of which were abnormal including 10 defined as ciTBI (Fig. 1). When we retrospectively applied the PR, 30/2613 patients were in the high-risk category, 653/2613 in the intermediate risk, and 1930/2613 in the low risk.

In the high risk, the PR recommends a CT scan and this was performed in 29/30 patients. In the intermediate risk group, the PR recommends observation or CT scan, and a CT scan was performed in 286/653 patients; in the low risk, the PR recommends no CT scan, but a CT scan was performed in 140/1930 patients.

In the intermediate risk group, 286/653 patients performed a head CT and 367/653 were observed: 13 were hospitalized in the ward, 354 were observed in the emergency department, with an average stay of 3.5 h.

Only 10 patients presented ciTBI, 7/10 underwent neurosurgery and 3/10 were hospitalized over two nights (Table 1). Among the 7/10 who underwent neurosurgery, applying the PR, 2 patients were classified in “CT scan recommended” category, the other 5 in “CT scan versus observation” and all presented clinical deterioration within the first hours of observation.

Concerning the 3/10 hospitalized patients with ciTBI, according to the PR, 2 were classified as a “CT scan recommended” category, and 1 in “CT scan versus observation” but, within the first hours of observation, he developed marked sleepiness.

Applying the PR, no patient with ciTBI would have been discharged without an accurate diagnosis, with a sensitivity of 100% (Table 1).

Regarding 40 patients with CT scan abnormalities, only 1 was considered by the PR “at low risk”, had a subarachnoid hemorrhage in the left frontal area, that did not require neurosurgery (Supplementary Table 1).

The statistical correlation between the PECARN recommendation categories and the presence of CT scan abnormalities was statistically significant (*p* = 0.000).

**Fig. 2:**
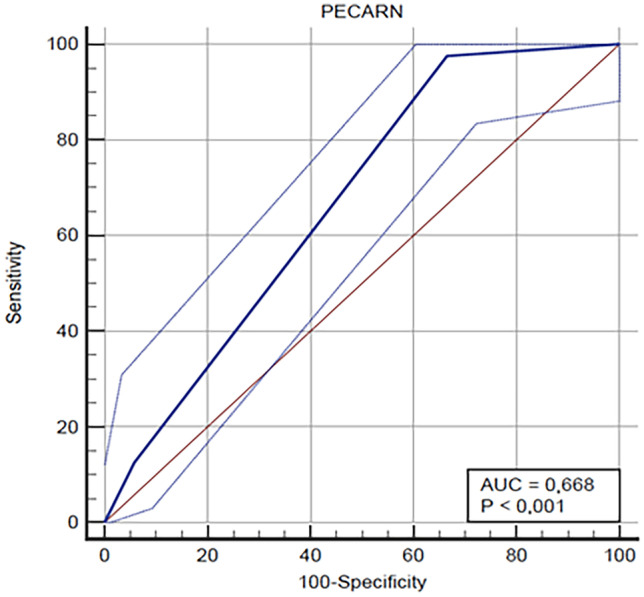
The ROC curve shows the specificity and sensitivity of the PECARN Rule in identifying children ≥ 2 years with CT scan abnormalities

The analysis of the ROC curves of the PR showed a sensitivity of 97.5% (CI 86.8–99.9%) in identifying patients with CT scan abnormalities and a specificity of 33.5% (CI 29–38.3%) (Fig. 2).

### Multivariate analysis

Multivariate analysis was performed to evaluate the single variable considered by the PR and their correlation with head CT scan abnormalities. We observed that repeated episodes of vomiting (OR: 6.0, CI: 1.2–6.3), a severe mechanism of trauma (OR: 3.4, CI: 1.6–7.1), and trauma in the parietal site (OR: 2.8, CI: 1.2–6.3) and in the occipital site (OR: 2.1, CI: 1.0–4.3) remain independently associated with head CT abnormalities.

**Under 2 years of age**- We included 1219 patients aged under 2 years, of whom 96/1219 (7.86%) received a CT scan of which 49/96 (51.04%) were abnormal including 3 defined as ciTBI (Fig. 1). Applying the PR, 17/1219 patients were in high-risk category, and 16/17 performed head CT scan (94.1%); 358/1219 in the intermediate risk category and 56/358 performed a head CT scan (15.6%). The remaining 844/1219 were in the low-risk category and 24/844 performed the CT scan (2.8%).

In the intermediate risk group, 56/358 children performed a head CT scan and 302/358 were observed: 9 were admitted to the ward and 293 were observed directly in the emergency department with an average stay of 3.14 h.

Only 3 patients presented ciTBI: 2/3 underwent neurosurgery and 1/3 was hospitalized more than two nights. Among the 2/3 who underwent neurosurgery, applying the PR, 1 patient was classified in “CT scan recommended” category and the other in the “CT scan versus observation” but few minutes after the beginning of clinical observation, the latter developed profound drowsiness and CT showed an epidural hematoma. The child hospitalized with ciTBI was classified in the “CT scan versus observation” category and presented clinical worsening and irritability for which he performed an intensive observation in PICU. Applying the PR also in this age group, no ciTBI would have been discharged without an accurate diagnosis with a sensitivity of 100% (Table 1).

Concerning the 49 patients with CT scan abnormalities, only one patient of the low-risk category presented CT scan abnormalities: right frontal subarachnoid hemorrhage that did not require neurosurgery (Supplementary Table 1).

Also in this age group, the statistical correlation between the PECARN recommendation categories and the presence of CT scan abnormalities was statistically significant (*p* = 0.000).

**Fig. 3:**
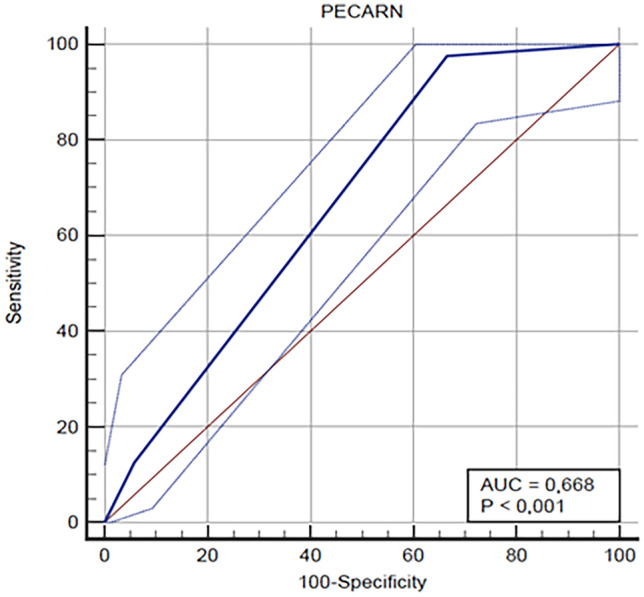
The ROC curve shows the specificity and sensitivity of the PECARN Rule in identifying children under 2 years with CT scan abnormalities

In children under 2 years, the analysis of the ROC curves of the PR showed a sensitivity of 97.96% (CI 89.1–99.9%) in identifying patients with CT scan abnormalities and a specificity of 48.94% (CI 34.1–63.9%) (Fig. 3).

### Multivariate analysis

In this age group at multivariate analysis, only the younger age at the time of trauma was independently associated with CT scan abnormalities; none of the other variables considered by the PR was independently associated.

## Discussion

Our study performed in an Italian pediatric ED confirmed the efficacy and safety of the PR in detecting the presence of ciTBI in patients with MHI, reducing radiological investigations and exposure to ionizing radiations. In literature, different authors proposed clinical algorithms to optimize management of pediatric patients with MHI but the PR seems to have the best methodological quality [6–16].

Despite the relatively low rate of performed CT scans in our ED, similar to other Italian [14] or American [15] centers, applying rigorously the PR we would have avoided 139 head CT scans in patients ≥ 2 years of age and 23 head CT scans in those < 2 years of age (29% less). This considers only patients in the “no CT scan” group according to the PR.

Analyzing the data in the intermediate risk category, the number of CT scans could be further reduced. In the population aged 2 years and older depending on how the intermediate recommendations are applied, among the 286 patients who underwent CT scan, only 34 had an abnormal CT scan and 6/34 presented ciTBI. In the population aged under 2 years, among the 56 patients who underwent CT scan, 40 had an abnormal CT scan and 2/40 with ciTBI. However, even in the “CT scan versus observation” category, the PR proved to be a useful tool. In fact, in this group, ED physicians should perform initial observation over CT scan, especially in the presence of isolated findings, no worsening of symptoms, and a child older than 3 months. Indeed, our results demonstrated that all 8 patients with ciTBI in this risk category had worsening of symptoms during clinical observation [9, 16].

This is also confirmed by a secondary analysis of the PECARN head injury parent study which showed that clinical observation before CT decision-making resulted in a safe and potentially effective strategy to manage a subset of children with MHI.

In our population, 653/2613 (25%) over 2 years and 358/1219 (29.4%) under 2 years belonged to the “CT scan versus observation” category. This may seem like a large number; however, the necessary observation can be carried out in the emergency department without the need of a pediatric ward, unless clinical worsening. In fact, it usually takes a few hours to understand the clinical evolution of the child. The PR suggests an observation of 4–6 h in this risk category except for the age group under 3 months in which greater caution is recommended.

In the population aged ≥ 2 years, we found from the multivariate analysis that vomiting showed a significant correlation with an abnormal CT scan. Vomiting is a frequent sign in children with head trauma (more than 20% of patients undergoing CT); this correlation is not demonstrated in the population aged < 2 years. Since our statistical analysis does not consider the number of episodes, we cannot consider this isolated sign as indicative of the presence of lesions in the CT scan. This is already supported by other authors [9, 15], in particular Dayan et al. in 2014 stated that TBI on CT is rare and clinically important TBI is very rare in children with minor blunt TBI when vomiting is their only sign or symptom [17].

In the population aged < 2 years, the only variable significantly correlated with positive CT was age, with an increased risk of having fracture or brain injury on CT as it decreases (*p* value = 0.02).

The retrospective application of the PR in our study showed a sensitivity of 100% in identifying patients with ciTBI in both age groups and very high negative predictive values in identifying patients as low risk, confirming the data already reported by other authors.

In our cohort of 3832 patients with MHI, only 13 had a ciTBI. In 4 of these patients, the rule recommended an immediate CT scan. None of the remaining patients belonged to the low-risk group. We also performed a subgroup analysis of patients who underwent CT scans following MHI. In our analysis, the statistical correlation between PECARN risk categories and the presence of fractures and/or intracranial lesions is statistically significant in both subgroups (*p* = 0.000).

Also considering patients with fracture or head injury, even if not clinically important, the PR demonstrated a sensitivity of 97.5% over 2 years and 97.9% under 2 years. One patient for each of the two age groups, having no clinical predictors of high risk for ciTBI, would have been discharged, even though without complications.

CT scan is the gold standard for the evaluation of patients with head trauma but it exposes children to carcinogenic ionizing radiation. Although nowadays the effective dose for head CT is low, the brain and red bone marrow doses are relatively high, especially in young children, resulting in the highest risks of brain cancer and leukemia [12, 13].

The study has some limitations. First of all, some patients were excluded because their parents refused observation in the ED or because there was a lack of data necessary for PR. However, we can postulate that they were equally distributed across the various PECARN risk categories without affecting the results.

Furthermore, among the patients who have not performed the CT scan, some may have had fractures or intracranial injuries that did not require surgery. If these patients were hospitalized for more than two nights, the number of ciTBIs in our study would have changed. To overcome this problem, we analyzed only patients who performed the CT scan, demonstrating a wide range of patients in which CT can be avoided safely.

Another limitation of the study is the absence of follow-up to rule out a second visit to another hospital for ciTBI. However, we believe this is unlikely to happen considering that the recommendations given to patients at discharge include returning to the ED if neurological symptoms appear.

The strengths of our study lie in the large number of patients enrolled and in the analysis of items of the PR individually. In fact, we can consider the parietal site and a severe mechanism of trauma as two red flags to be carefully considered in the group over the age of 2 years. On the other hand, under the age of two, attention needs to be increased particularly in younger children, as indeed underlined by the rule.

In many cases, even if the clinician knows the guidelines, he does not apply them because he is not convinced of their validity and efficacy, in particular in pathologies such as head trauma that can have serious sequelae and medico-legal implications if not adequately diagnosed and treated. In these cases, clinicians rely on their experience and while knowing the latest guidelines on the subject, they tend to carry out more diagnostic tests for the “safety” of the child.

The PR is already considered a useful and safe tool for the management of children presenting to the ED with MHI; in our setting, we found 100% sensitivity in both age groups in identifying patients with ciTBI. Therefore, in patients classified in the low-risk category, it is the duty of the physician not to expose the child to ionizing radiation.

Furthermore, analyzing the subpopulation of patients who have performed the CT scan, the rule has proved to be extremely sensitive also in identifying patients with fractures or TBI even if not clinically important.

Since the beginning of our retrospective study, we have tried to implement adherence to the guidelines based on the PECARN Rule through training lessons, printed material, and flow charts hanging on the walls of the visit rooms of the pediatric emergency department.

Currently, PECARN Rule is used routinely in the case of pediatric head trauma. We think that this strategy, combined with periodic reports on clinical practice, could be valid tools to implement guideline adherence and consequently minimize exposure to ionizing radiation.

Finally, we can conclude that by applying the PR, unnecessary CT scans can be safely reduced in favor of more clinical observations and reduced radiation exposure.

## Supplementary Information

Below is the link to the electronic supplementary material.

Supplementary file1 (TIF 360 KB)

Supplementary file2 (DOCX 17 KB)

## Data Availability

N/A.

## Abbreviations

ciTBI: Clinically important traumatic brain injury
CT: Cranial computed tomography
ED: Emergency department
IQR: Interquartile range
MHI: Minor head injuries
PR: PECARN Rule

## Code availability

N/A.
