# Supplementary figures and images for: PECARN Rule in diagnostic process of pediatric patients with minor head trauma in emergency department
Source: Eur J Pediatr. 2022 Feb 22;181(5):2147–54. doi: 10.1007/s00431-022-04424-9 (PMC9056473; doi:10.1007/s00431-022-04424-9)

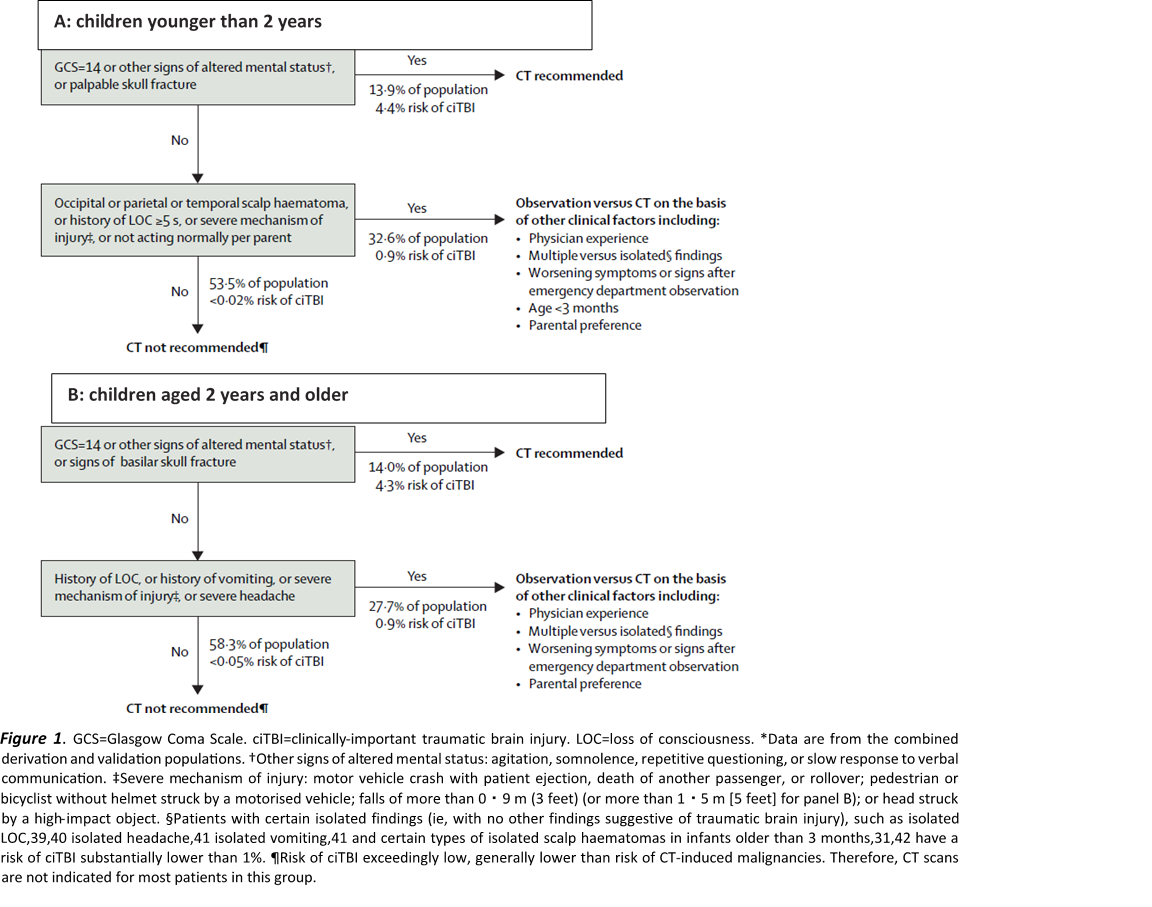

Supplement: Supplementary file 1 — Supplementary file1 (TIF 360 KB) [file 431_2022_4424_MOESM1_ESM.tif]
