# Supplementary material for: PECARN Rule in diagnostic process of pediatric patients with minor head trauma in emergency department
Source: Eur J Pediatr. 2022 Feb 22;181(5):2147–54. doi: 10.1007/s00431-022-04424-9 (PMC9056473; doi:10.1007/s00431-022-04424-9)
Supplement: Supplementary file 2 — Supplementary file2 (DOCX 17 KB) [file 431_2022_4424_MOESM2_ESM.docx]

| Age group | <2 years | ≥ 2 years |
| --- | --- | --- |
| Abnormal CT scan (n patients) | 49 | 40 |
| CT recommended  Skull fracture  Rupture of Arteriovenous Malformation | 8 (16.3%)  8  0 | 5 (12.5%)  4  1 |
| CT versus observation  Skull fracture  Sub-arachnoid haemorrhage  Sub-dural haematomas | 40 (81.6%)  39  1  0 | 34 (85.0%)  28  4  2 |
| CT not recommended  Sub-arachnoid haemorrhage | 1 (2.1%)  1 | 1 (2.5%)  1 |

**Supplementary Table 1**
